# A novel bacterium-like particles platform displaying antigens by new anchoring proteins induces efficacious immune responses

**DOI:** 10.3389/fmicb.2024.1395837

**Published:** 2024-05-22

**Authors:** Lingdi Niu, Mingchun Gao, Hongkun Ren, Xinqi De, Zhigang Jiang, Xinyao Zhou, Runhang Liu, Hai Li, Haoyuan Duan, Chuankun Zhang, Fang Wang, Junwei Ge

**Affiliations:** ^1^Heilongjiang Provincial Key Laboratory of Zoonosis, College of Veterinary Medicine, Northeast Agricultural University, Harbin, China; ^2^National Key Laboratory for Animal Disease Control and Prevention, Harbin Veterinary Research Institute, Chinese Academy of Agricultural Sciences, Harbin, China

**Keywords:** bacterium-like particles, anchoring protein, surface display platform, multi-epitope antigen, immunogenicity

## Abstract

Bacterium-like particles (BLP) are the peptidoglycan skeleton particles of lactic acid bacteria, which have high safety, mucosal delivery efficiency, and adjuvant effect. It has been widely used in recent years in the development of vaccines. Existing anchoring proteins for BLP surfaces are few in number, so screening and characterization of new anchoring proteins are necessary. In this research, we created the OACD (C-terminal domain of *Escherichia coli* outer membrane protein A) to serve as an anchoring protein on the surface of BLP produced by the immunomodulatory bacteria *Levilactobacillus brevis* 23017. We used red fluorescent protein (RFP) to demonstrate the novel surface display system’s effectiveness, stability, and ability to be adapted to a wide range of lactic acid bacteria. Furthermore, this study employed this surface display method to develop a novel vaccine (called COB17) by using the multi-epitope antigen of *Clostridium perfringens* as the model antigen. The vaccine can induce more than 50% protection rate against *C. perfringens* type A challenge in mice immunized with a single dose and has been tested through three routes. The vaccine yields protection rates of 75% for subcutaneous, 50% for intranasal, and 75% for oral immunization. Additionally, it elicits a strong mucosal immune response, markedly increasing levels of specific IgG, high-affinity IgG, specific IgA, and SIgA antibodies. Additionally, we used protein anchors (PA) and OACD simultaneous to show several antigens on the BLP surface. The discovery of novel BLP anchoring proteins may expand the possibilities for creating mucosal immunity subunit vaccines. Additionally, it may work in concert with PA to provide concepts for the creation of multivalent or multiple vaccines that may be used in clinical practice to treat complex illnesses.

## 1 Introduction

A prevalent intestinal illness in chicken production, necrotizing enteritis (NE) is mostly brought on by a *Clostridium perfringens* type A infection (Song et al., 2022). The disease has a significant financial impact on the world’s poultry market (Wade et al., 2015). *C. perfringens* could produce a variety of toxins (Uzal et al., 2010, 2014), and the main virulence factors of *C. perfringens* type A causing enteritis are alpha toxin, NetB, and CPE, of which alpha toxin is the most lethal (Li et al., 2013). Antibiotics are currently the main treatment for NE. However, they have a lot of negative effects. As of right now, there are no commercially available vaccines for full protection against NE (Kulkarni et al., 2007; Jiang et al., 2015; Wilde et al., 2019; Shamshirgaran et al., 2022). The only commercial vaccine now available for necrotic enteritis is broiler hens immunized with an alpha toxoid generated from a type A strain of *C. perfringens* (NetvaxH) in order to provide maternal protection in chicks (Crouch et al., 2010), and the use of this traditional vaccine to prevent and control NE remains risky. There is an urgent need to find alternative solutions to problems such as increased incidence of NE (Kulkarni et al., 2007; Prescott et al., 2016; Razmyar et al., 2017). The subunit vaccinations have several advantages, including good specificity, reduced side effects, and greater safety. In order to maximize the effectiveness of most subunit vaccinations, immunopotentiators must be added, such as influenza vaccines (Khalaj-Hedayati et al., 2020), COVID-19 vaccines (Mekonnen et al., 2022), and mink virus vaccines (Wang et al., 2021), etc.

Bacterium-like particles (BLPs) are mostly prepared from lactic acid bacteria (LAB), from which hollow peptidoglycan (PG) skeleton particles found in cell walls are retained after proteins and nucleic acids are extracted using heat and acid treatment. BLP has the characteristics of adjuvants and vaccine carriers via TLR2-mediated signaling. The PG of LAB can efficiently elicit innate immune responses (Bosma et al., 2006; Li et al., 2019). BLP is a type of immunopotentiator with enormous potential in vaccine development (Heine et al., 2015). Because of their high safety, mucosal delivery efficiency, and adjuvant effect, BLP is widely used in the production of mucosal adjuvants and mucosal vaccines (Zhang et al., 2022; Su et al., 2023; Wang et al., 2023; Zhang et al., 2023). There is a need to expand the range of mucosal vaccines based on BLP because there are currently few available due to the high technical requirements. BLPs obtained from different immunomodulatory LABs have different adjuvant capacities when used as vaccine adjuvants (Raya Tonetti et al., 2020), but most of the existing studies on BLPs are prepared from *Lactococcus lactis*, only a few studies have used the probiotic *Levilactobacillus* to prepare BLP (Zhou et al., 2023). BLPs need an anchoring domain to anchor the antigens to their surface. The transmembrane anchoring, lipoprotein anchoring, LPXTG, LysM, WxL, and S-layer proteins are examples of the traditional anchoring domains (Visweswaran et al., 2014; Mao et al., 2016; Michon et al., 2016; Desvaux et al., 2018). The most used covalent anchoring protein in the BLP surface display system is the LPXTG of the *Streptococcus pyogenes* M6 protein (Dieye et al., 2001; Kylä-Nikkilä et al., 2010), and the non-covalent anchoring protein is a protein anchor (PA) protein consisting of three LysM sequences (Steen et al., 2005; Andre et al., 2008; Okano et al., 2008). Although several anchoring proteins have been identified, there are still a few that have been applied to the surface display system of LAB, so it is vital to screen for and find new anchoring proteins.

In our previous study (Jia et al., 2024), when we bound the unpurified soluble PA fusion protein expressed in *Escherichia coli* (*E. coli*) to BLPs and verified it by SDS-PAGE, we found there is another *E. coli* protein that can also bind to BLPs in addition to the PA fusion protein. By searching the Pfam database, we found that the C-terminal domain of *E. coli* outer membrane protein A (OmpA) (PF00691) can bind to PG through non-covalent anchoring. Therefore, we hypothesized that the C-terminal domain of *E. coli* OmpA is expected to be a new anchoring protein to display antigens on the surface of BLPs. OmpA is one of the main outer membrane proteins of *E. coli*. The OmpA-like domain is found in the C-terminal region of many Gram-negative bacterial outer membrane proteins in molecular biology. The α helix of the *E. coli* C-terminal domain is strikingly comparable to the previously published PG binding domain of *Neisseria meningitidis* RmpM (Grizot and Buchanan, 2004), and this part of the protein might interact with the PG layer. Ishida et al. further identified the PG binding site of *E. coli* OmpA (Ishida et al., 2014), but this discovery has not yet been implemented in the surface display system.

This study aimed to determine whether the *E. coli* OmpA C-terminal domain may serve as an alternative anchoring protein in the BLP surface display system for the development of a novel *C. perfringens* vaccine. The effectiveness of the new surface display system was validated using red fluorescent protein (RFP) as the signal protein. The surface display platform was utilized to exhibit the multi-epitope antigen of *C. perfringens* (CPMEA) and assess its immunogenicity. To this end, (i) we predicted the functional domains of the C-terminal domain of OmpA (named OACD) through bioinformatics analysis and used OACD as an anchor to bind to BLPs made from *Levilactobacillus brevis* 23017 to construct the OACD-BLP23017 surface display system. (ii) The effectiveness, stability, and compatibility of the novel surface display system were identified using RFP as a signal protein. (iii) CPMEA as the model antigen was displayed on the surface of BLP23017 to construct a vaccine (COB17). The vaccine COB17 was administered through different routes in the mice model, and the immunogenicity was evaluated by observing changes in body weight, bacterial protection rate, systemic immune response-related antibodies, mucosal secretory IgA, inflammatory factors, and intestinal pathological changes. (iv) Two anchoring proteins—OACD and PA—are employed to display distinct proteins on a BLP to investigate their capacity to simultaneously show adjuvants and antigens.

## 2 Materials and methods

### 2.1 Bacterial strains and growth conditions

*Levilactobacillus brevis* 23017 (CCTCC AB 2018164), *Levilactobacillus plantarum* 27197, and *Levilactobacillus casei* 27382 were isolated and identified by our laboratory to have excellent probiotic activity (Cui et al., 2018; Shi et al., 2019). The standard strains *Levilactobacillus acidophilus* ATCC4356, *Levilactobacillus rhamnosus* LGG, *Lactococcus lactis* MG1363, *Bacillus subtilis* WB800N, and *C. perfringens* strain C57-1 (contain alpha toxin) were stored in our lab. The *E. coli* CVCC230 isolate was purchased from the China Institute of Veterinary Drug Control (Beijing, China). MRS medium was used to cultivate *Levilactobacillus* at 37°C. At 30°C, *L. lactis* MG1363 was cultivated in an M17 medium. The LAB strains were incubated in a constant-temperature incubator for 18–24 h until the OD_600_ value reached 1 and were used for subsequent experiments. *C. perfringens* C57-1 was cultured anaerobically in cooked meat medium at 37°C in a constant-temperature incubator overnight, then adjusted the concentration to 10^8^ CFU/200 μL. *B. subtilis* WB800N and *E. coli* CVCC230 were cultured in LB medium at 37°C in a constant-temperature incubator overnight for use in PCR.

### 2.2 PCR amplification of OACD and construction of fusion protein

We used the PDB database,^1^ to look up the C-terminal domain of *E. coli* OmpA, mapped its protein molecular structure with Pymol,^2^ and analyzed surface electrostatic potential.

**Figure 1:**
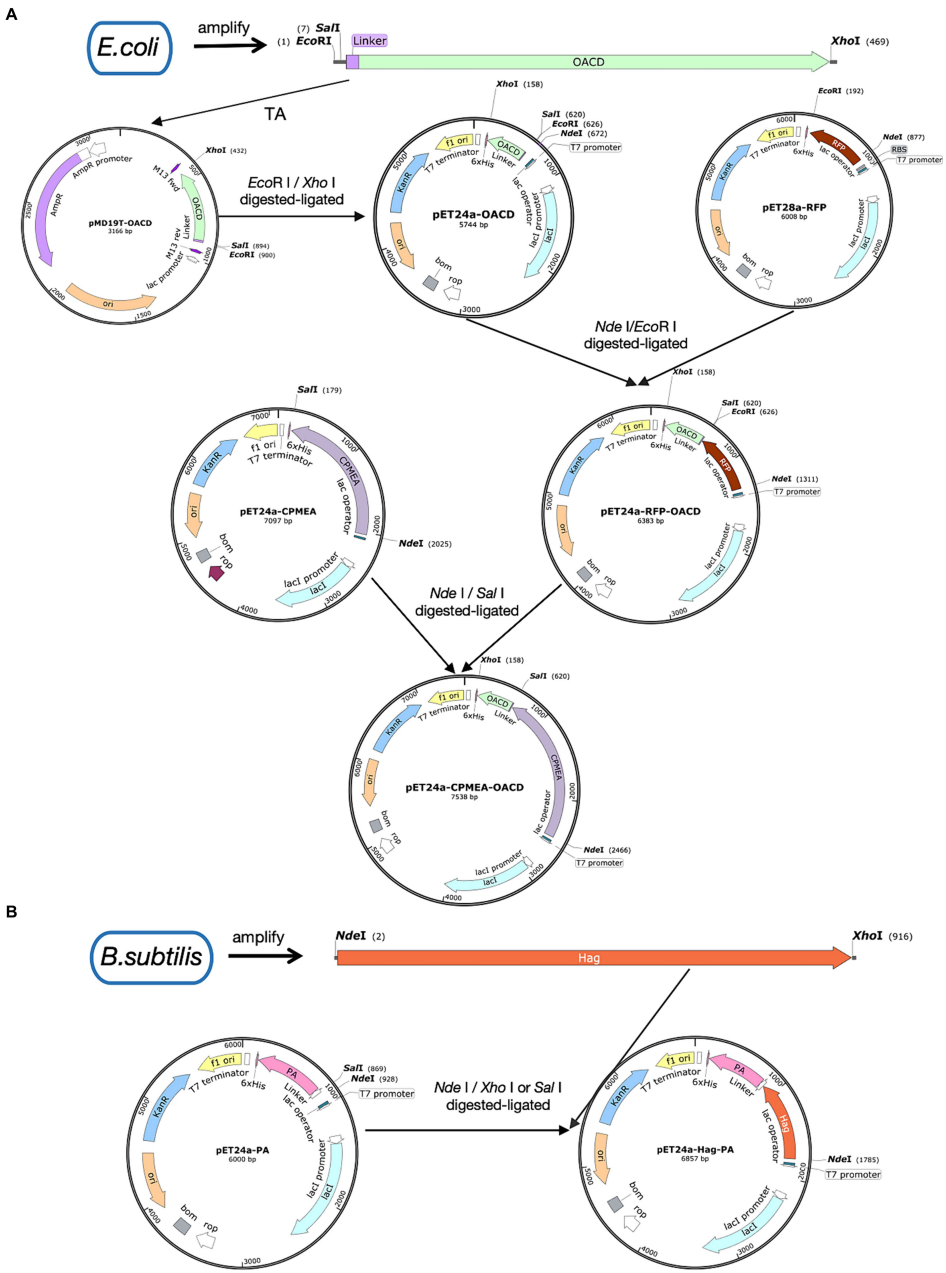
Illustration of plasmid construction. Plasmid maps were made by SnapGene. **(A)** The procedure for the construction of the pET24a-CPMEA-OACD plasmid. **(B)** The procedure for the construction of the pET24a-Hag-PA plasmid.

Figure 1A shows the process of plasmid construction. The C-terminal domain of *E. coli* OmpA was named OACD. *E. coli* CVCC230 strain was used as the PCR template to amplify OACD as the anchoring protein with the primers in Supplementary Table S1, the N-terminus contains the *Eco*R I site and the C-terminus contains the *Xho* I site. The amplified OACD fragment was linked to the pMD™19-T vector by the cloning kit (3,271, Takara, Beijing, China), followed by inserted into the expression plasmid pET-24a at the *Eco*R I (1040A, Takara, Beijing, China) and *Xho* I (1094A, Takara, Beijing, China) sites using T4 DNA ligase (2011A, Takara, Beijing, China) according to the instructions. The constructed plasmid was pET24a-OACD. The plasmid pET28a-RFP has been saved in our laboratory. At the same time, the RFP fragment was inserted into the N-terminal of OACD at the *Nde* I (1161A, Takara, Beijing, China) and *Eco*R I sites, named pET24a-RFP-OACD. The recombinant plasmids were transformed into *E. coli* Rosetta will select positive clones for recombinant protein expression. The positive clones were expanded and cultured in 5 mL LB liquid medium at 37°C with shaking at 180 rpm. Until their OD600 value reached 0.6, followed by a 13 h induction by isopropyl β-D-1-thiogalactopyranoside (IPTG, 1 mM) (I6070D, Biotopped, Beijing, China) at 22°C with shaking at 180 rpm. The absorbances were measured at 600 nm with a spectrophotometer (SpectraMax ABS & ABS PLUS, Molecular Devices, Shanghai, China). The induced bacteria were collected through centrifugation and broken by ultrasound at 200 W for 20 min in ice-water. After sonication and centrifugation of the expressed bacteria, the obtained supernatant contains the soluble protein.

### 2.3 Bacterium-like particles

As previously mentioned (Fu et al., 2018), BLPs were prepared using chemical pretreatment. Freshly cultured LAB strains were suspended in 10 mL of 10% trichloroacetic acid and boiled in a water bath for 45 min. Next, the particles were washed three times with 25 mL PBS to remove nucleic acids and proteins. After the last wash step, the particles were resuspended with PBS and stored at −80°C. The BLPs, which were made from *L. lactis* MG1363, *L. brevis* 23017, *L. plantarum* 27197, *L. acidophilus* ATCC4356, *L. rhamnosus* LGG, and *L. casei* 27382, were, respectively, named BLP1363, BLP23017, BLP27197, BLP4356, BLPLGG, and BLP27382. To verify whether RFP can bind to BLPs, the prepared BLPs were added to the supernatant containing the RFP-OACD fusion protein and shaken slowly at 100 rpm for 30 min at 37°C. To remove unbound proteins, the particles were washed with PBS three times. Finally, the binding product was resuspended with sterile PBS.

### 2.4 Fluorescence microscopy

According to the reference (Gordillo et al., 2020), we can verify whether RFP-OACD successfully binds to BLPs by directly observing whether there was a red light on the surface of BLPs by ultra-high Resolution (Deltavision OMX SR, GE, Fairfield, CT, USA). To this end, we aspirated 2 μL fusion product onto microscope slides and promptly covered them with coverslips. We sealed the perimeter of the coverslips with colorless nail polish. Finally, the resulting slides were observed at ultra-high Resolution.

### 2.5 Stability of the proteins bound to BLPs

According to the literature (Raya-Tonetti et al., 2021), we explored the stability of OACD bound to BLPs. The products of RFP-OACD bound to BLP23017 were, respectively, washed with different molar concentrations of NaCl (0.2 M, 0.5 M, and 1 M), urea (0.5 M, 1 M, and 2 M), and buffers of different pH values (4, 7, and 9). Finally, each fusion product was fixed at OD600 of 1, and the RFU of each sample was measured by a fluorescence spectrophotometer (F-7100, HITACHI, Tokyo, Japan). Also, we determined the binding ability of RFP-OACD to BLP23017 at different temperatures (4°C, 30°C and 37°C). The experiment was conducted in three replicates.

### 2.6 *Clostridium perfringens* Multi-epitope antigen bound to BLP

Bioinformatics analysis is increasingly being used to create multiple-epitope vaccines that are non-allergenic, non-toxic, and free of unwanted peptide fragments. In our previous experiments, bioinformatics analysis was used to predict the dominant antigenic epitopes of several proteins of *C. perfringens* to construct the multi-epitope group antigen CPMEA. It includes dominant antigenic epitopes such as Net_B146-322_, alpha toxin_284-398_, and Zmp_698-1022_, targeting different toxins secreted by *C. perfringens* (Guo et al., 2024). These predicted dominant epitopes were linked with five peptide antigens and five B-cell epitopes, which have been shown to be immunogenic in existing studies (Duff et al., 2019; Aldakheel et al., 2021), to provide protection against various toxins. We used the restriction sites of *Nde* I and *Sal* I to replace the RFP in pET24a-RFP-OACD with CPMEA. Finally, pET24a-CPMEA-OACD was transformed in *E. coli* Rosetta by heat shock. Subsequently, positive clones were screened and induced for expression using the 2.2 mentioned method. After sonication and centrifugation, the supernatant containing CPMEA-OACD was purified using the His-tag Protein Purification Kit (P2229S, Beyotime, Shanghai, China). Then, the purified supernatant was mixed with BLP23017 to prepare COB17.

### 2.7 Anchoring different antigens to BLP surface

To demonstrate that OACD and PA might co-display multiple antigens on the BLP surface, we amplified the flagellin protein Hag of *Bacillus subtilis* and linked it to PA. This flagellin protein of *Bacillus subtilis* possesses potent immunoadjuvant functions. Figure 1B shows the process of plasmid construction. *Bacillus subtilis* WB800N was used as the PCR template to amplify the Hag gene, the N-terminus contains the *Nde* I site and the C-terminus contains the *Xho* I site. PET24a-PA with *Nde* I and *Sal* I (1080A Takara, Beijing, China) sites has been successfully constructed in previous studies. Because *Sal* I and *Xho* I are isocaudomers, the enzymatic sites of *Nde* I and *Xho* I/*Sal* I can be utilized to insert Hag into pET24a-PA to eventually obtain the recombinant plasmid pET24a-Hag-OACD. Positive clones were expressed with the method mentioned in 2.2, then purified using the His-tag Protein Purification Kit. The purified soluble CPMEA-OACD protein and Hag-PA protein were well mixed and bound to BLP23017 by shaking the mixture slowly at 100 rpm for 30 min at 37°C. To remove unbound proteins, the particles were washed with PBS three times. Finally, the binding product was resuspended with sterile PBS. Identification of two proteins bound to BLP by SDS-PAGE analysis of the resuspended binding product.

### 2.8 SDS-PAGE and Western blot analysis

SDS-PAGE and Western blotting were used to analyze the expression and purification of CPMEA-OACD and Hag-PA proteins, as well as the proteins bound to BLPs. Briefly, the recombinant protein was transferred to a polyvinylidene difluoride (PVDF) membrane following SDS-PAGE. The membrane was blocked with 5% powdered skim milk at 37°C for 2 h. Then, wash three times with PBS and incubate with the primary antibody at 4°C overnight. Western blot analysis of CPMEA-OACD using the anti-α-toxoid antibody diluted at 1:1000 as the primary antibody (Ys-2273R, YaJi biological, Shanghai, China) and a horseradish peroxidase (HRP)-conjugated goat anti-rabbit IgG antibody (Sigma, United States) diluted at 1:2000 as the secondary antibody. The western blot analysis of Hag-PA using the His Tag Mouse Monoclonal Antibody (HRP Conjugated) (1:2000; AF2873, Beyotime, Shanghai, China). After washing, the enhanced chemiluminescence (ECL) (BL520A, biosharp, Beijing, China) was used to detect the conjugated horseradish peroxidase. The relative expression of specific bands was analyzed and quantified using ImageJ Software (GeneGnome XRQ, Syngene, United Kingdom).

### 2.9 Immunization and infection

4–6 weeks old female Kunming mice with weight of 18–22 g were purchased from Changsheng Biological Company (Liaoning, China). The mice were maintained in a controlled environment and had free access to rodent food and tap water during a 12-h cycle of light and darkness. Before the formal experiment, the mice were allowed to adapt to the laboratory environment for 3 days. To explore the best route to inoculate COB17, the mice were randomly divided into five groups (n = 8), and each group was immunized by different immune routes. On the d 0, the control and infection groups were subcutaneous injected with PBS 200 μL. The next three groups were immunized with the same doses of COB17 but via different routes, subcutaneous immunization (50 μg/200 μL); intranasal immunization (50 μg/100 μL), the COB17 was slowly dropped into the nasal cavity of mice and inhaled naturally (50 μL/nostril), and 30 min between immunizations of both nostrils; and oral immunization (50 μg/200 μL). Blood and feces from mice were collected on d 7, 25, and 37 after immunization. The blood was obtained from the tail vein and centrifuged at 1,800 × g for 10 min, and the serum was taken for the cytokines and antibodies test. Briefly, fecal samples as weighted pellets were added to PBS containing 0.1% sodium azide (1 mL/100 mg fecal sample). The pellet was vortex mixed and centrifuged at 13,000 × g for 10 min at 4°C. Supernatants were collected and stored at −70°C for the determined of IgA antibodies according to the method of reference (Kim et al., 2021). On the 31st d of immunization, mice other than those in the control group were challenged with *C. perfringens* type A (10^8^ CFU/200 μL) by intraperitoneal injection. After the infection, weight changes and survival rates of mice were observed and recorded for 7 days. Finally, all mice were euthanized by cervical dislocation to collect jejunum and spleen tissues. The spleen tissues were stored in −80°C for RNA extract. Intestinal mucus from the jejunum of mice was collected for total secretory immunoglobulin A (SIgA) content. The collected ileum tissues were fixed in 10% formalin pathological section. The experiment was conducted in three replicates. The animal study protocol was approved by the Institutional Review Board of the Northeast Agricultural University for animal experiments (NEAUEC-20200303).

### 2.10 Detection of antibody level

Based on Zhang et al.’s method (Zhang et al., 2022), specific antibodies against *C. perfringens* type A (IgA, IgG) were detected by indirect ELISA. Briefly, 96 well plates (31,121, Labselect, Tianjin, China) were coated with 3.4 μg/mL CPMEA 100 μL per well overnight at 4°C, then blocked for 2 h at 37°C with 5% skim milk. The serum of mouse collected on d 7, 25, and 37 was diluted 1:200 and then serially diluted at a 2-fold ratio, added serum to 96 well plates, and incubated at 37°C for 1.5 h. Peroxidase-bound anti-mouse IgG (A0216, Beyotime, Shanghai, China) or IgA (RS030211, Immunoway, Texas, United States) antibodies were added and incubated at 37°C for 1 h. The reaction was carried out with a TMB (PR1210, Solarbio, Beijing, China) substrate reagent. The enzyme reaction was stopped after 20 min by the addition of 2 M H_2_SO_4_, and the optical density was measured at 450 nm with a spectrophotometer (Thermo Fisher Scientific, New York, NY, USA). High-affinity antibody responses were measured by indirect ELISA as described above after addition of urea (4 mM) to remove antibodies bound to the antigen with low affinity (Kim et al., 2021). The presence of a high affinity antibody indicates that the antibody is more specific and can validate the accuracy of the specific antibody results. The mucosal SIgA was detected by ELISA kit according to the instructions (D721136, Sangon Biological Technology, Shanghai, China). The experiment was conducted in three replicates.

### 2.11 Detection of cytokines

Based on the method of Shi et al. (2022), the detection of spleen tissue cytokines was implemented with a little modification. The spleen tissues of mice were collected aseptically, and total RNA was extracted from the spleen using the RNA extraction kit (ER101-01, TransGen Biotech, Beijing, China). Then reverse transcribed into cDNA using ReverTra Ace® (TRT-101, TOYOBO, Shanghai, China), following the manufacturer’s protocol, each 40 μL reverse transcription reaction contained 2 μg RNA template. The cDNA was used for quantification and expression with 2 × Hi SYBR Green qPCR Mix (A2250B, HaiGene Biotech Co., Ltd., Harbin, China). Furthermore, the Real-Time Quantitative PCR reactions were carried out using the Applied Biosystems® 7,500 Real-Time PCR Systems (Analytik Jena AG, Jena, Germany) according to the instructions. Actin β was taken as the reference gene. Primers for this study were synthesized with the company (Comate Bioscience Co., Ltd., Changchun, Jilin, China) and provided in Supplementary Table S1 according to the reference (Cheng et al., 2014; Chen et al., 2016; Pan et al., 2022; Zhang and Yi, 2022). The relative mRNA levels were quantified using the 2^-ΔΔ*CT*^ method (Livak and Schmittgen, 2001). The experiment was conducted in three replicates.

Serum IL-4 (EK0405), IL-5 (EK0408), TNF-ɑ (EK0527), and TGF-β1(EK0515) cytokine levels were detected by ELISA kit according to the instructions (Boster Biological Technology, China). The experiment was conducted in three replicates.

### 2.12 Toxin neutralization test

The method of toxin neutralization test *in vitro* was determined according to the method that has been reported (Goossens et al., 2016). The supernatant of *C. perfringens* C57-1 cultured anaerobically in cooked meat medium was cultured overnight on the blood plate at 37°C. It was observed that the internal complete hemolytic ring caused by θ-toxin and the external incomplete hemolytic ring caused by α-toxin appeared on the blood plate. We mixed the supernatant of *C. perfringens* C57-1 with the serum of the mice immunized in equal volumes and incubated at 37°C for 2 h. The mixture was dropped on the blood plate and evaluated for hemolytic situation after overnight culture. The experiment was conducted in three replicates.

### 2.13 Histopathology

According to the previous established pathological tissue staining protocol (Trajković et al., 2007), after 7 d of *C. perfringens* C57-1 challenged, the mouse ileum were collected and fixed in 10% paraformaldehyde at room temperature for at least 48 h for histopathological evaluations. Pathological sections were prepared by a commissioned company (Wuhan Servicebio Technology Co., Ltd., Wuhan, Hubei, China).

### 2.14 Statistical analysis and software

All data were statistically analyzed using GraphPad Prism 9 software. Multiple comparisons, compare the mean of each column with the mean of every other column, of one-way ANOVA were used to analyze the significance of differences between groups. Survival analysis was performed using the Kaplan–Meier method, and differences in the survival rates were assessed by the log-rank test. Results are the averages from independent experiments with standard deviations indicated by error bars. Results were expressed as mean with SD. The asterisks above bars refer to differences compared to the control group; the two groups connected by a horizontal line with asterisks above them represent the differences compared between these two groups. Statistical significance of *p* < 0.05 indicates a significant difference, and *p* < 0.01 indicates a highly significant difference. The primers designed in this study were done with oligo7 software.

## 3 Results

### 3.1 The discovery and structural analysis of the C-terminal of *E. coli* OmpA as a new anchoring domain

**Figure 2:**
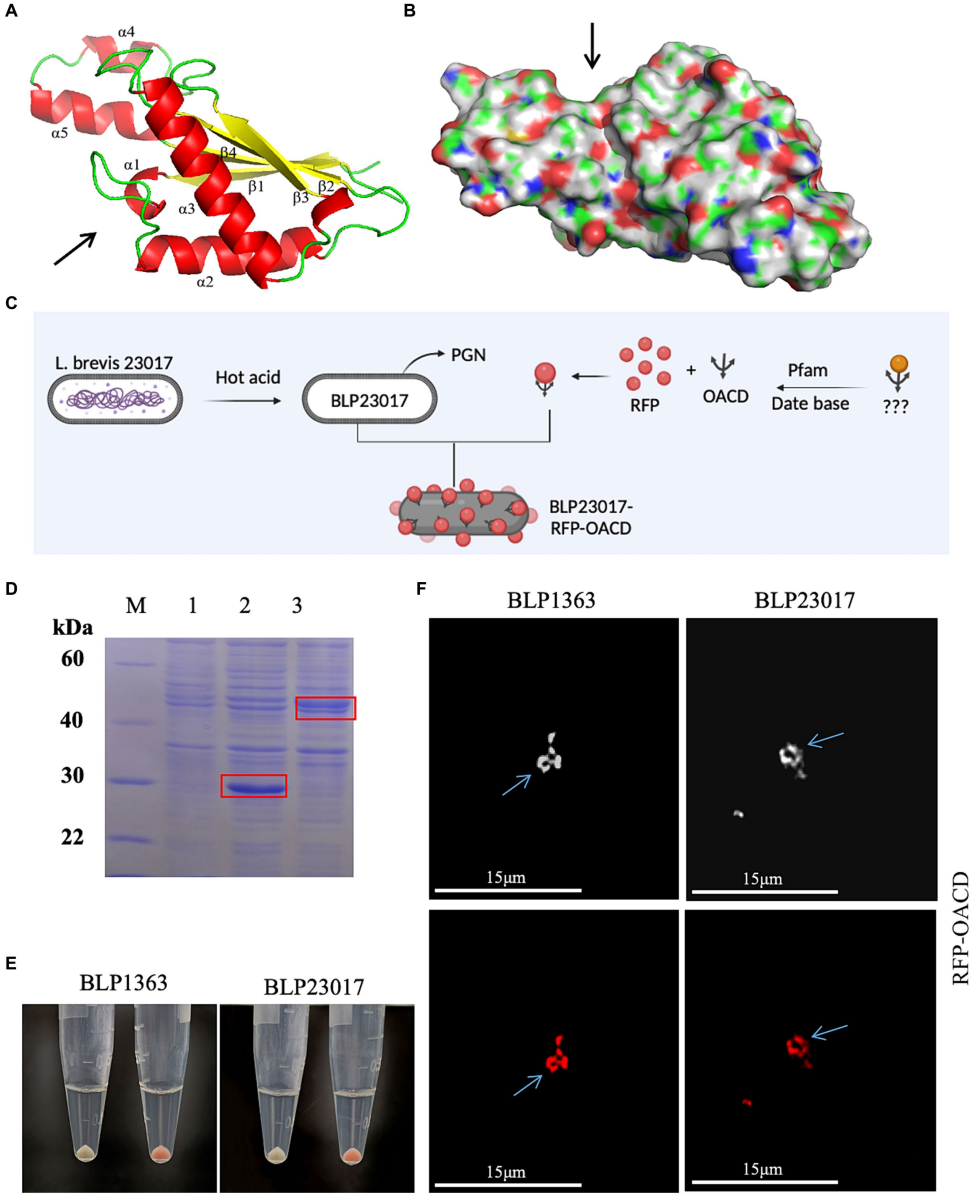
**(A)** The structure of the C-terminal domain of *Escherichia coli* (*E. coli*) outer membrane protein A. α chain is represented by red, β chain is indicated by yellow, the arrow indicates the direction of chains, and the presumed peptidoglycan (PG) binding point is indicated by the arrow. **(B)** The hydrophilic sink that can accommodate the PG chain is indicated by the arrow. **(C)** Flow chart of BLP surface displaying red fluorescent protein (RFP). Created with BioRender.com. **(D)** Identified the expression of RFP and RFP-OACD by SDS-PAGE. M: Protein marker; 1: Negative sample; 2: Expressed RFP protein; 3: Expressed RFP-OACD proteins. **(E)** RFP and BLP incubation as well as RFP-OACD and BLP incubation were observed in bacteria color by centrifugation of the bacteria, and the bacteria in which RFP was successfully bound to BLP showed red coloration. The white precipitate of bacteria indicates the RFP did not bind to BLP1363 (left panels) or BLP23017 (right panels). The red precipitate of bacteria indicates the binding products of RFP-OACD with BLP1363 (left panels) and BLP23017 (right panels). **(F)** Fluorescence microscopy observation of the binding products of RFP-OACD with BLP1363 and BLP23017. Cells were observed by white light (up panels) and red light (down panels).

Figures 2A,B depicts the structure of the C-terminal of *E. coli* OmpA. The C-terminal domain of OmpA consists mainly of two long α chains and four β chains, which is known as “β/α/β/α-β” fold. β4 chain and other β chains are reverse parallel, with short helix α1 connecting β1 and α2, and the extension formed by α4 and α5 is fixed by a disulfide bond. According to the literature, we indicated the presumed PG binding point with an arrow. The results of surface electrostatic potential showed that the environment of the groove indicated by the arrow was very hydrophilic, and it was speculated that the hydrophilic groove could accommodate PG chains.

### 3.2 RFP display on the surface of BLP1363 and BLP23017 by OACD

We identified if OACD can serve as an innovative anchoring protein for displaying antigens on the BLP surface based on the flowchart in Figure 2C. Firstly, RFP protein and RFP-OACD fusion protein were constructed and expressed, and the purified proteins were used to bind to BLPs. As shown in Figure 2D, we characterized protein expression by SDS-PAGE. After three washes of PBS, the precipitate after RFP without OACD bound to BLPs had no color, and the precipitate after RFP-OACD bound to BLPs was red. This result indicates that the binding of RFP and BLPs is dependent on OACD (Figure 2E). In addition, the phenomenon observed by fluorescence microscopy further confirms that RFP was bound to BLPs by OACD (Figure 2F).

### 3.3 OACD stability combined with the surface of BLP23017

The products of RFP-OACD bound to BLP23017 were washed with buffer solutions having varying molar concentrations of NaCl, urea, and pH in order to determine the stability of the OACD-BLP23017 surface display system. As shown in Supplementary Figure S1A, the samples were washed with NaCl buffer solutions of different concentrations, and OACD still adheres to the surface of BLPs. Similarly, when washing with urea of different concentrations, OACD still adheres heavily to BLP, and higher concentrations of urea may be required for separation. At pH = 4, it was observed that fluorescence intensity was stronger. As the pH value increased, the fluorescence intensity gradually decreased. At the same time, we also bound the fusion protein to BLPs at different temperatures (4°C, 30°C, 37°C). At 30°C and 37°C, the binding ability of OACD to BLPs was not significantly different. At low temperatures, the binding capacity is significantly reduced. Overall, these results confirm that the binding of OACD to BLP is relatively stable. In summary, these results confirm that the OACD-BLP23017 surface display system is stable.

### 3.4 OACD combined to the surface of a variety of BLPs

BLPs were produced and bound to RFP-OACD in order to investigate the possibility of using OACD as an anchoring protein for the presentation of antigens on various LAB surfaces. As shown in Supplementary Figure S1B, the binding product of RFP-OACD bound to BLPs after washing and centrifugation showed a red precipitate, indicating that OACD can bind to BLP27197, BLPLGG, BLP4356 and BLP27382 prepared from different LABs.

### 3.5 CPMEA display on the surface of BLP23017 by OACD

**Figure 3:**
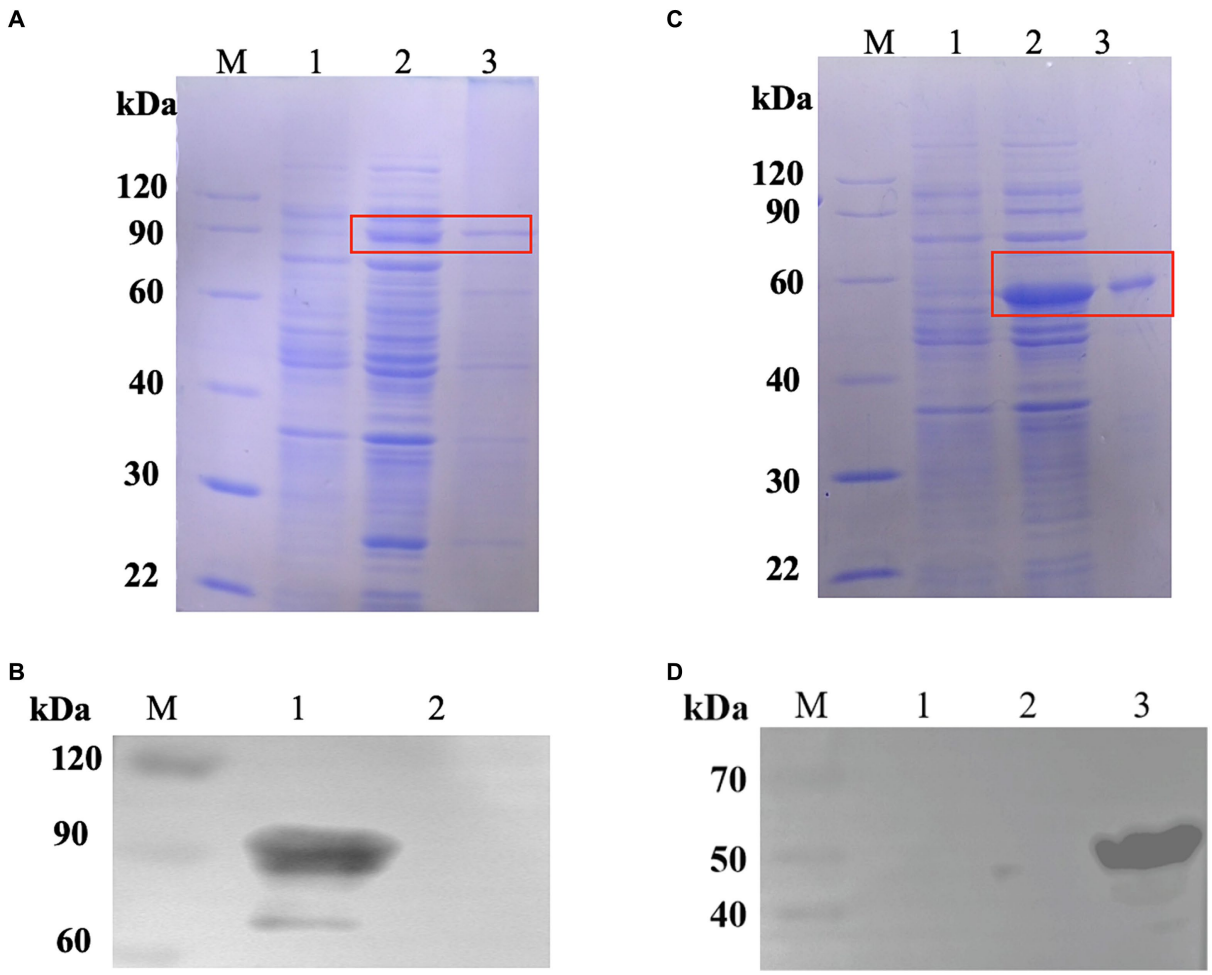
Expression of fusion proteins. **(A)** Identification of the protein CPMEA-OACD by SDS-PAGE. M: Protein marker; 1: Noninduced bacteria; 2: Supernatant after sonication; 3: Soluble protein after CPMEA-OACD purification. **(B)** Identification of the protein CPMEA-OACD by western blot. M: Protein marker; 1: Supernatant after sonication; 2: Negative control. The anti-α-toxoid antibody (1:1000) is the primary antibody, and a horseradish peroxidase (HRP)-conjugated goat anti-rabbit IgG antibody (1:2000) is the secondary antibody. **(C)** Identification of the protein Hag-PA by SDS-PAGE. M: Protein marker; 1: Noninduced bacteria; 2: Supernatant after sonication; 3: Soluble protein after Hag-PA purification. **(D)** Identification of the protein Hag-PA by western blot. M: Protein marker; 1: Negative control; 2: negative control plasmid pET24-a; 3: Supernatant after sonication. The His Tag Mouse Monoclonal Antibody (HRP-conjugated) was used for western blot analysis of Hag-PA.

We created the pET24a-CPMEA-OACD recombinant plasmid in order to create the COB17 vaccine. Under the induction of IPTG at 22°C overnight, CPMEA-OACD achieved optimal expression. SDS-PAGE (Figure 3A) and western blot (Figure 3B) detected similar bands with predicted molecular weights of CPMEA-OACD. Western blot analysis used the anti-α-toxoid antibody (1:1000) as the primary antibody and a horseradish peroxidase (HRP)-conjugated goat anti-rabbit IgG antibody (1:2000) as the secondary antibody. Next, CPMEA-OACD is bound to BLP23017. SDS-PAGE confirmed that the fusion protein was successfully bound to BLP23017, which demonstrates the successful construction of COB17 (Supplementary Figure S2).

### 3.6 Different antigens display on the BLP surface by PA and OACD

To demonstrate that OACD and PA might co-display multiple antigens on the BLP surface, we constructed pET24a-Hag-PA. SDS-PAGE (Figure 3C) and Western blot (Figure 3D) detected similar bands with predicted molecular weights of Hag-PA. The purified CPMEA-OACD protein and Hag-PA protein were co-incubated with BLP23017 and analysis by SDS-PAGE of bound BLP particles. The results showed that the co-incubation group of both proteins with BLP showed similar bands with predicted molecular weights of CPMEA-OACD and Hag-PA proteins. It confirmed the successful binding of the CPMEA-OACD protein and Hag-PA protein with BLP23017 (Supplementary Figure S2). Finally, multiple antigens were successfully displayed on the surface of the same BLP by OACD and PA.

### 3.7 Single immunization with COB17 by different immunization routes has a good immune protective effect

**Figure 4:**
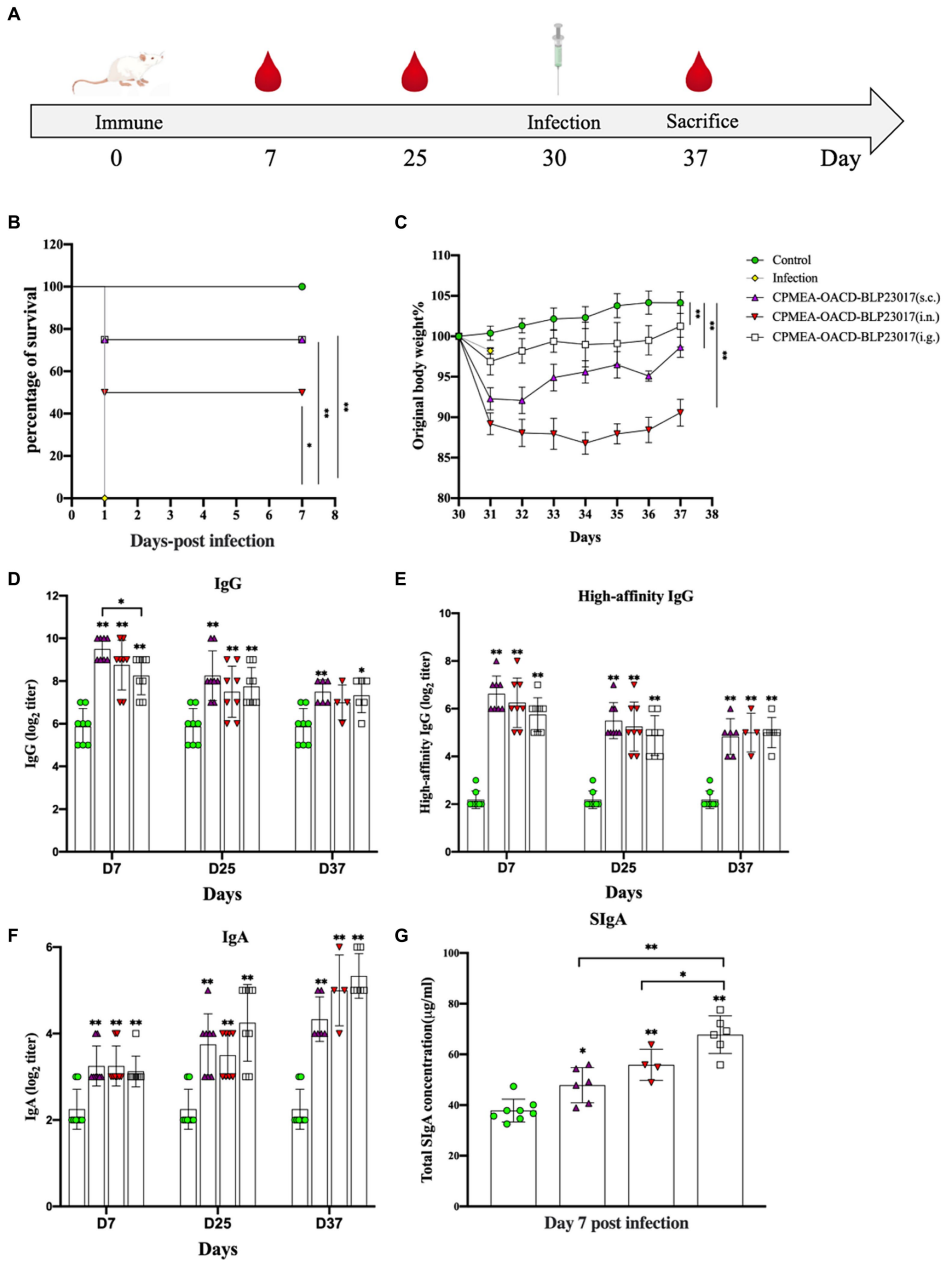
**(A)** The experimental protocol of mouse immunization. **(B)** Curves showing the survival of the mice. The abscissa represents days, the ordinate represents the survival rate of mice. **(C)** The curves of body weight changes in mice. The abscissa represents days, and the ordinate represents the percentage of body weight per mouse per day after the infection relative to the initial body weight (body weight of the mouse the day before the infection). **(D)** Serum specific IgG antibody levels. **(E)** Serum high-affinity IgG antibody levels. **(F)** Fecal specific IgA antibody levels. An indirect ELISA was performed to detect *Clostridium perfringens* specific antibodies (IgG, high affinity IgG and IgA). Purified CPMEA protein was used as the antigen used in the detection of mouse model-specific antibody levels. **(G)** SIgA antibody levels in intestinal mucus. It was tested according to the instructions of the mouse secretory immunoglobulin A (SIgA) ELISA kit. Results are the averages from independent experiments with standard deviations indicated by error bars. The asterisks above bars refer to differences compared to the control group; the two groups connected by a horizontal line with asterisks above them represent the differences compared between these two groups. *p < 0.05, **p < 0.01.

The challenge assay using *C. perfringens* C57-1 was used to evaluate the immune-protective effect of COB17 in 8 mice per group (Figure 4A). Figure 4B demonstrates the survival rate, in which all mice in the infection group died on the first day. In the three immunized groups, the survival rates were significantly higher compared to the infection group (*p* < 0.05). The immune protection rate was 75% (6/8) in the CPMEA-OACD-BLP23017 (s.c.) group, 50% (4/8) in the CPMEA-OACD-BLP23017 (i.n.) group, and 75% (6/8) in the CPMEA-OACD-BLP23017 (i.g.) group. Figure 4C demonstrates the percent change in body weight. All mice in the immunized group had significantly lower body weights compared to the control group on the seventh day after the infection (*p* < 0.01). The mice in the CPMEA-OACD-BLP23017 (i.g.) group showed a minimal weight loss trend, and the CPMEA-OACD-BLP23017 (i.n.) group showed the largest trend. The above results showed that CPMEA-OACD-BLP23017 had a good immune protective effect on *C. perfringens* C57-1 infected mice, and the oral immunization group had a better immune effect.

### 3.8 COB17 Enhanced antibody levels in mice

To evaluate the antibody levels in the serum of COB17-induced mice, specific IgG and high-affinity IgG antibodies were determined in serum samples. As shown in Figures 4D,E, the specific IgG antibodies and the levels of high-affinity IgG antibodies were significantly higher in all vaccination groups than in the control group (*p* < 0.01). The above results also showed that serum IgG antibody levels were higher in the subcutaneous injection group.

The serum-specific IgA and SIgA antibody levels were determined to evaluate the level of mucosal immunity induced by COB17 in mice. Figure 4F showed that the specific IgA antibodies were significantly higher in all vaccination groups than in the control group (*p* < 0.01). Figure 4G shows that the total SIgA of the three immunized groups of mice was higher than that of the control group. The above results also showed higher levels of mucosal immunity in the oral immunization group.

### 3.9 COB17 regulated the production of cytokines

**Figure 5:**
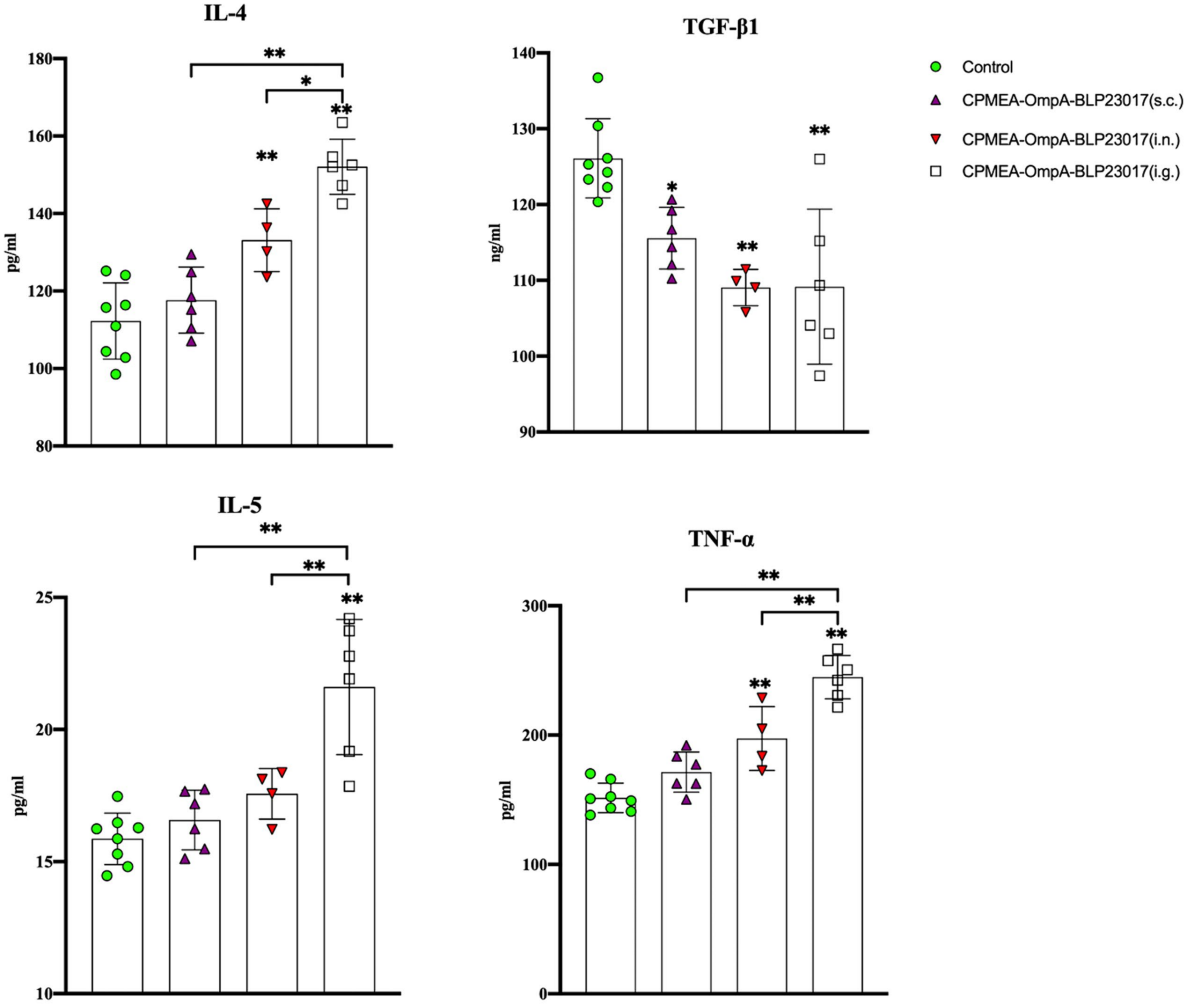
Serum IL-4, TGF-β1, IL-5, and TNF-ɑ cytokine levels were detected by ELISA kit. The ordinate represents the cytokine concentration in serum. Results are the averages from independent experiments with standard deviations indicated by error bars. The asterisks above bars refer to differences compared to the control group; the two groups connected by a horizontal line with asterisks above them represent the differences compared between these two groups. ^*^p < 0.05, ^**^p < 0.01.

To assess the changes in immune-related factors in serum, we measured levels of IL-4, IL-5, TNF-α, and TGF-β1 by ELISA kits. Figure 5 shows that the levels of IL-4, IL-5, and TNF-α in each immunization group were higher than the control group, while the results in TGF-β1 were the opposite. The results indicate that the oral COB17 vaccine significantly regulates the expression of immune-related factors in serum.

**Figure 6:**
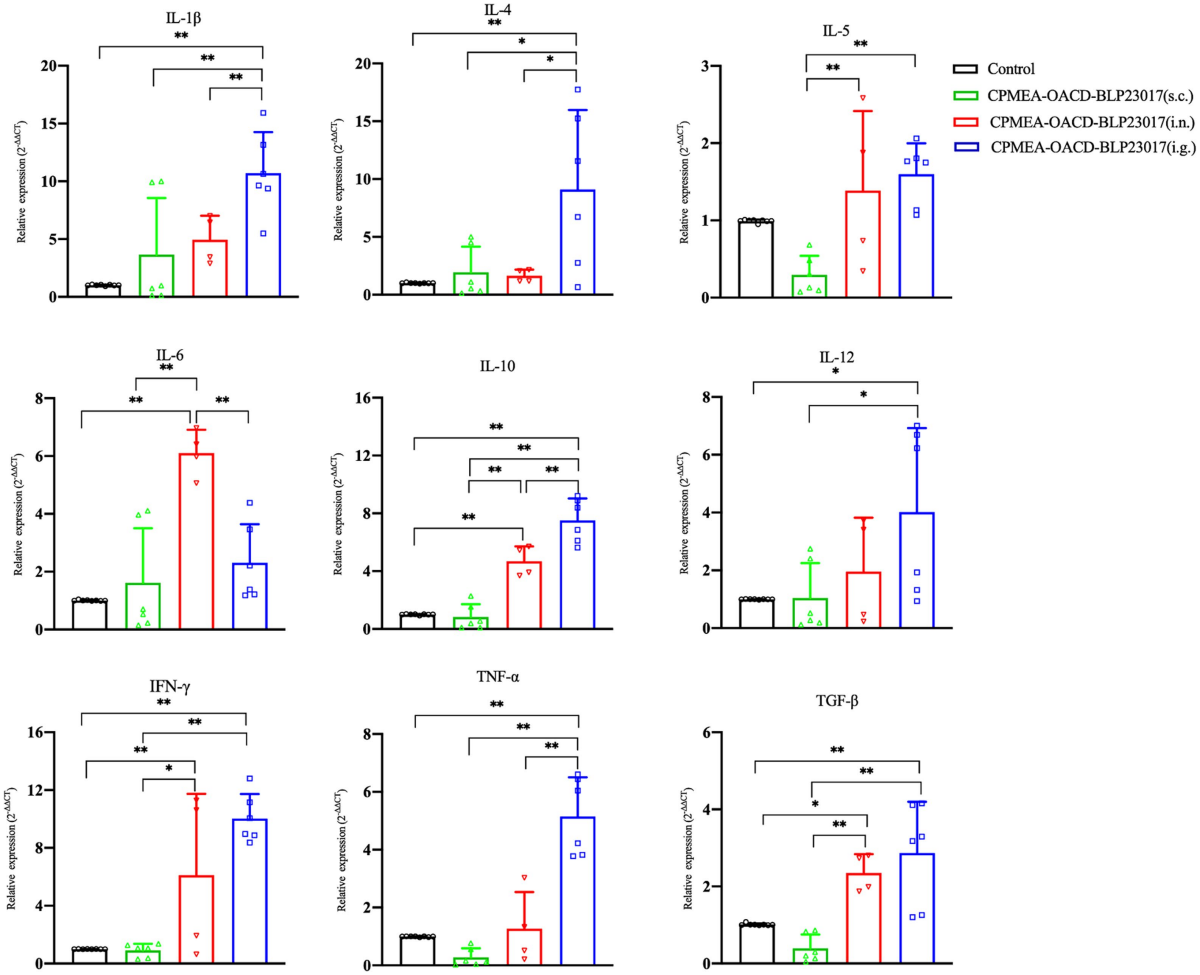
Gene transcription of spleen immune related cytokine. Real-time qPCR reactions were used for detection, and β-action was seen as reference gene. The abscissa represents groups, the ordinate represents 2^−ΔΔ*CT*^ value. Results are the averages from independent experiments with standard deviations indicated by error bars. The two groups connected by a horizontal line with asterisks above them represent the differences compared between these two groups. ^*^p < 0.05, ^**^p < 0.01.

To determine the changes in immune-related cytokines in splenocytes, we performed real-time qPCR analysis of the mRNA expression. Figure 6 depicts the levels of each cytokine in the CPMEA-OACD-BLP23017 (i.g.) and CPMEA-OACD-BLP23017 (i.n.) groups were higher than the control group. The CPMEA-OACD-BLP23017 (s.c.) group had higher levels of IL-1β, IL-4, IL-6, and IL-12 than the control group. These results indicate that the oral COB17 vaccine significantly increased the expression of immune-related cytokines in splenocytes compared to subcutaneous and intranasal drops.

### 3.10 COB17 improves resistance to the challenge of alpha toxin

**Figure 7:**
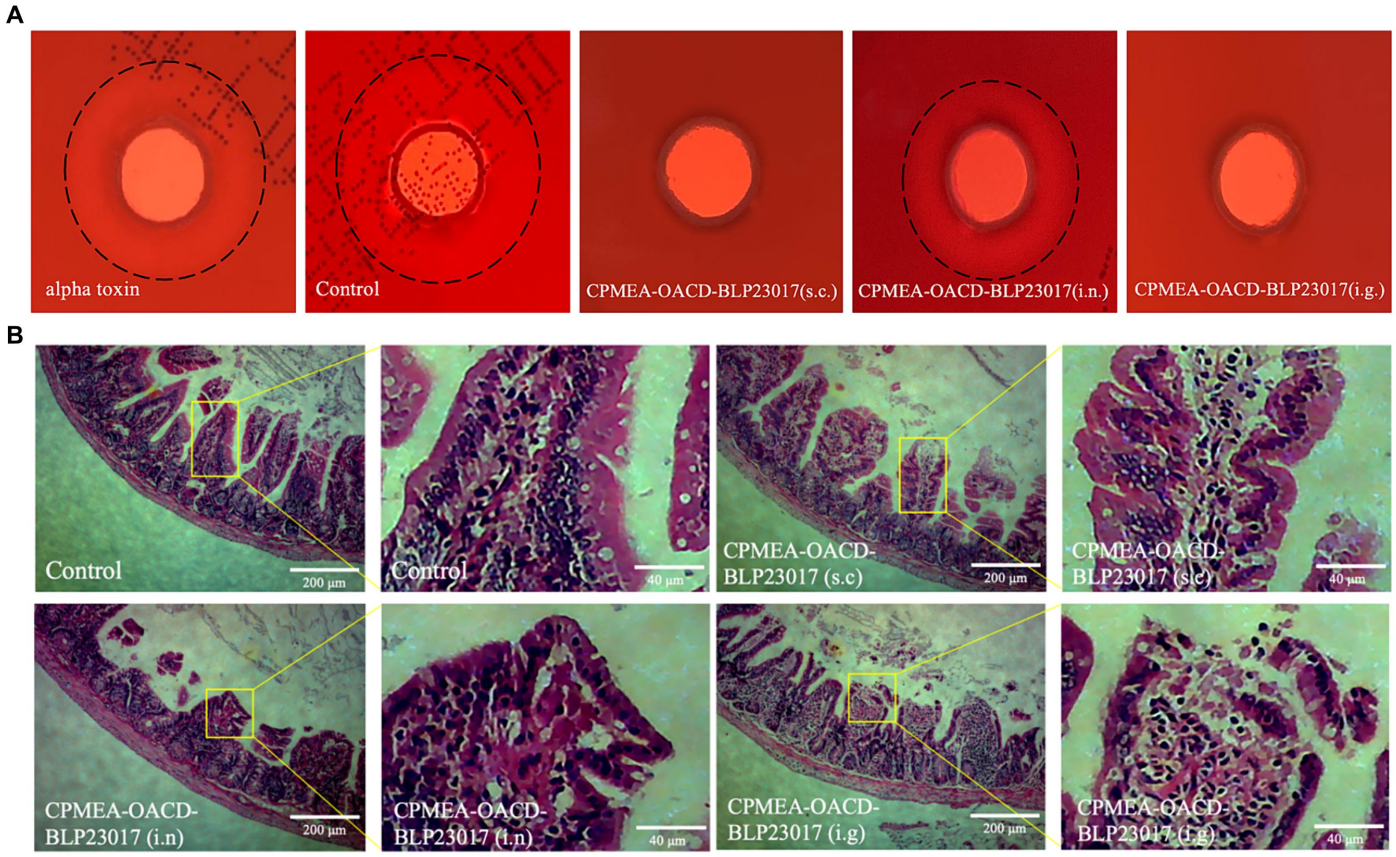
**(A)** Experimental results of neutralizing toxin in mouse serum *in vitro.* The black dashed line represents the size of the hemolysis ring. **(B)** The pathological changes of ileum tissue induced by the immunization after challenge. The pathological changes were examined by HE staining, the lesion location has been enlarged.

To demonstrate that mice immunized with COB17 can be neutralize to alpha toxin infection in addition to *C. perfringens* type A infection, we performed alpha toxin neutralization assays *in vitro* using sera collected from the immunized mice. As shown in Figure 7A, an external hemolytic ring was visible on the blood plate after the control group sera were co-incubated with the alpha toxin, which size does not differ much from the size of the external incomplete hemolytic ring caused by the alpha toxin. The CPMEA-OACD-BLP23017 (i.n.) group showed a slight larger hemolytic ring compared to the other immunized groups, indicating that this group of mice was less protected against alpha toxin challenge compared to the other immunized groups. In conclusion, the mice in the three immunization groups were able to neutralize the challenge of alpha toxin.

### 3.11 COB17 protect the intestinal tissue integrity after infection

To observe the intestinal damage of *C. perfringens* type A in mice, the ileum was collected and prepared into pathological sections. As shown in Figure 7B, the ileum in the control group shows a normal morphology with neat and complete villi and no inflammatory cell infiltration. The submucosa and epithelial cells of the ileum in the CPMEA-OACD-BLP23017 (s.c.) group were normal, with only slight inflammatory cell infiltration. The ileum of the CPMEA-OACD-BLP23017 (i.n.) group had submucosal edema with the detachment of epithelial cells and severe inflammatory cell infiltration. The ileum in the CPMEA-OACD-BLP23017 (i.g.) group had normal mucosal epithelial cell structure, a regular and neat arrangement, and slight inflammatory cell infiltration. The results showed that COB17 can help prevent the intestinal damage induced by *C. perfringens* type A in mice.

## 4 Discussion

Subunit vaccines offer several benefits, but one drawback that cannot be overlooked is their poor immunogenicity without a suitable adjuvant. Scientists usually use it together with immunopotentiators when preparing vaccines to improve immunogenicity (Khalaj-Hedayati et al., 2020; Wang et al., 2021; Mekonnen et al., 2022). Studies have confirmed that BLP is an effective immunopotentiator, and the BLP surface display platform has also been applied to the development of vaccines for various diseases (Zhang et al., 2022; Su et al., 2023; Wang et al., 2023; Zhang et al., 2023). There are very few anchoring proteins available for the BLP surface display platform, though. In order to improve the selectivity of anchoring proteins, we built a stable surface display method for OACD-BLP23017 and found and identified OACD, a new anchoring protein. In addition, we have demonstrated that OACD can be widely applied to most LAB strains. We also used CPMEA as a model antigen to evaluate the good immunogenicity of the OACD-BLP23017 surface display system, which carried the antigen through three immunization routes. In conclusion, the OACD-BLP23017 surface display system developed in this work showed that *C. perfringens* multi-epitope antigens have good mucosal immunogenicity, offering suggestions for *C. perfringens* treatment, control, and prevention.

The technique by which the C-terminal domain of *E. coli* OmpA was identified as a novel anchoring protein in this study is intriguing. We can search for more stable anchoring proteins that can trigger higher immunogenicity by using this method to identify other anchoring proteins that can be connected to BLP or other bacterial surfaces. Although several domains have been identified as having the ability to non-covalently, only a few have been applied to LAB’s surface display systems, and the commonly used PA protein is patented. Attempts have been made to find new anchoring proteins to display proteins on the BLP surface. Domains that have been reported as new anchoring proteins include the C-terminal domain of *Levilactobacillus* acidophilus SlpA (Gordillo et al., 2020), the LysM domain of Pgb, and the Acglu proteins from *Levilactobacillus* fermentum (Raya-Tonetti et al., 2021). According to reports, *Pseudomonas aeruginosa* OprF’s C-terminal domain interacts with PG. Additionally, OprF’s amino acid sequence is 39% identical to that of *E. coli* OmpA’s C-terminal domain (De Mot and Vanderleyden, 1994; Koebnik, 1995; Ishida et al., 2014). Additionally, some studies that have demonstrated the ability of the C-terminal domain of *E. coli* OmpA to form a structurally separate entity that has a significant fraction of α-helix secondary structure (Vogel and Jähnig, 1986; Sugawara et al., 1996; Danoff and Fleming, 2011). This protein fragment is in the periplasm and can interact with the PG layer (De Mot and Vanderleyden, 1994; Koebnik, 1995). The PG-associated lipoprotein (PAL) and other previously identified PG-associated domains bear striking structural similarities (Parsons et al., 2006) of *Haemophilus influenzae* and *Neisseria meningitides* RmpM (Grizot and Buchanan, 2004). The length of OACD was shorter than that of the commonly used PA protein, and the fusion protein formed is more readily soluble for expression. On this basis, we verified that the C-terminal domain of *E. coli* OmpA could be used as a new and better anchoring protein in the BLP surface display platform. In order to improve the anchoring of exogenous proteins to the surface of BLP, the OACD was non-covalently attached to the surface as a novel anchoring protein, enriching the variety of anchoring proteins on the BLP surface display platform.

We showed that OACD can be applied universally to most LAB strains and that it is stable under simulated gastrointestinal pH, bile salts, and protease conditions by building a stable OACD-BLP23017 surface display system employing the novel anchoring protein and BLP derived from *L. brevis* 23017. This fact indicates that it has a protective effect against adverse conditions in the gastrointestinal tract (Gordillo et al., 2020; Raya-Tonetti et al., 2021). When the pH value changes, the binding between OACD and BLP23017 decreases as the pH value increases. The stable BLP expression system could be maintained at room temperature without the requirement for cold chain transit, significantly lowering vaccination costs (Bosma et al., 2006). Furthermore, the universality of the OACD-mediated surface display mechanism is demonstrated by the fact that it can function in various *levilactobacilli*.

We demonstrated distinct proteins on the BLP surface that separately transported by PA and OACD. Flagellin functions as a powerful immunological adjuvant. The immunostimulatory and inflammatory properties of *Bacillus subtilis* Hag recombinant flagellin have been evaluated. Some studies have shown that the vaccine based on Hag recombinant flagellin induced strong M2e-*specific* antibody responses and Th1/Th2-type immune responses. Overall, recombinant flagellin from *Bacillus subtilis* can be used as an adjuvant to reduce the level of inflammatory factors and side effects while retaining potent immunostimulatory properties (Côté-Cyr et al., 2022). We showed that the two anchoring proteins may act simultaneously without interfering with one another, and we proposed a unique idea for the production of multivalent vaccines by successfully associating CPMEA and Hag with the BLP using the new anchoring protein and PA protein.

According to the study’s findings, mice were inoculated with COB17 using three different methods, each of which was capable of eliciting a robust immune response after a single immunization, and oral immunization induced better mucosal immune responses, leading to more efficient protection. The OmpA protein has 325 residues, can mediate *E. coli* virulence and pathogenicity, and it has become a significant target in the immune response (Krishnan and Prasadarao, 2012). It is highly immunogenic (Confer and Ayalew, 2013) and may elicit both innate and adaptive immunological responses in the host (Novinrooz et al., 2017). Vaccines designed based on BLP can be administered by different routes (van Roosmalen et al., 2006; Ramirez et al., 2010; Saluja et al., 2010; Nganou-Makamdop et al., 2012; Van Braeckel-Budimir et al., 2013), but different routes of administration will lead to different protection rates. Furthermore, the issue with BLP-based vaccinations is that they need to be given again or more than once to improve immune responses, which lengthens the immunization period and raises the expense of vaccination. In this study, the COB17 vaccine constructed based on OACD anchoring proteins can induce strong systemic immune responses in mice with a single immunization and can achieve a protection level of more than 50% under the challenge of *C. perfringens* type A.

In comparison to other immunological pathways, intranasal immunization with BLP-based vaccines has been shown in multiple investigations to be less effective at eliciting systemic immunity (Zhang et al., 2019; Bi et al., 2020), and intranasal immunization enhanced mucosal immunity in addition to producing a systemic immune response (de Haan et al., 2012; Rigter et al., 2013; Li et al., 2018). These are all similar to the results in this study. The induction of an antigen-specific mucosal immune response requires a suitable adjuvant or delivery system (Czerkinsky et al., 1999). Results from the study of serum and spleen cytokines showed that the levels of cytokines in the subcutaneous immunization group were lower. Our results show that COB17 can achieve good immune effects regardless of the method of immunization, and oral immunization can induce better mucosal immunity. The developed OACD-BLP23017 surface display system exhibits the ability to promote mucosal immunization of the OACD-BLP23017 surface display system, making it a viable antigen delivery system for subunit vaccine research.

The display of recombinant proteins on bacterial surfaces is an area of research that is still in its infancy but has a wide range of potential biotechnological applications. Now, any protein or peptide might theoretically be attached to a PG-encapsulated bacterium, according to anchoring protein (Visweswaran et al., 2014). The expression of heterologous proteins on the surface of LAB has been used to develop mucosal vaccinations (van Roosmalen et al., 2006; Berlec et al., 2013; Van Braeckel-Budimir et al., 2013; de Azevedo et al., 2015) for the treatment of disease. We discovered and identified a new anchoring protein in this study, but we did not contrast it with the known anchoring protein. The constructed COB17 vaccine was only studied in mice for immunogenicity and not in broilers. In the next study, we will compare the binding capacity of OACD and commonly used anchoring proteins bound to BLPs, and the constructed COB17 vaccine will be studied in broiler chickens. According to the COB17 vaccine prepared in this study, one immunization in mice can achieve a good immune effect and cause good mucosal immune responses. The developed OACD-BLP23017 surface display system can be applied to unit vaccination research as a novel antigen display platform. The investigation of multivalent vaccinations for complicated diseases in clinical practice can also benefit from the surface presentation of numerous antigens on BLP.

## 5 Conclusion

In this study, we discovered and identified the C-terminal domain of *E. coli* OmpA as a new anchoring protein that can anchor onto the surface of BLP prepared by *L. brevis* 23017 to construct the effective, stable, and mucosal immunogenic OACD-BLP23017 surface display system. Additionally, OACD can be co-anchored to a variety of LAB surfaces, which sets the stage for future development of multiple types of BLP vaccines. We used this OACD-BLP23017 surface display platform to construct a *C. perfringens* vaccine with good immunogenicity. In subsequent studies, it is meaningful to use the OACD-BLP surface display platform for the production of multivalent BLP mucosal immunization vaccines to protect against multiple pathogenic infections that can be induced simultaneously.

## Data availability statement

The raw data supporting the conclusions of this article will be made available by the authors, without undue reservation.

## Ethics statement

The animal study was approved by The Ethical Committee of the Institute approved all scientific experiments. All applicable international and national guidelines for the care and use of animals in experiments were followed. Approval (NEAUEC-20, 3 March 2020) was obtained from the Institutional Committee of Northeast Agricultural University for animal experiments. The study was conducted in accordance with the local legislation and institutional requirements.

## Author contributions

LN: Writing – original draft, Data curation, Formal analysis, Methodology. MG: Writing – review & editing, Conceptualization, Formal analysis, Funding acquisition, Investigation, Resources, Validation. HR: Writing – original draft, Data curation, Methodology, Validation. XD: Writing – original draft, Methodology, Validation. ZJ: Formal analysis, Methodology, Validation, Writing – original draft. XZ: Writing – original draft, Data curation, Formal analysis, Software. RL: Writing – original draft, Data curation, Software, Validation. HL: Writing – original draft, Data curation, Software. HD: Writing – original draft, Formal analysis, Data curation, Software. CZ: Writing – original draft, Data curation, Methodology, Software. FW: Writing – review & editing, Conceptualization, Data curation, Formal analysis, Funding acquisition, Investigation, Methodology, Project administration, Resources, Software, Supervision, Validation, Visualization, Writing – original draft. JG: Writing – review & editing, Conceptualization, Data curation, Formal analysis, Funding acquisition, Investigation, Methodology, Project administration, Resources, Software, Supervision, Validation, Visualization, Writing – original draft.

## Footnotes

^1^ https://www.rcsb.org/

^2^ https://pymol.org/2/
