# Corrigendum: A novel bacterium-like particles platform displaying antigens by new anchoring proteins induces efficacious immune responses

**DOI:** 10.3389/fmicb.2025.1625053

**Published:** 2025-05-23

**Authors:** Lingdi Niu, Mingchun Gao, Hongkun Ren, Xinqi De, Zhigang Jiang, Xinyao Zhou, Runhang Liu, Hai Li, Haoyuan Duan, Chuankun Zhang, Fang Wang, Junwei Ge

**Affiliations:** ^1^Heilongjiang Provincial Key Laboratory of Zoonosis, College of Veterinary Medicine, Northeast Agricultural University, Harbin, China; ^2^National Key Laboratory for Animal Disease Control and Prevention, Harbin Veterinary Research Institute, Chinese Academy of Agricultural Sciences, Harbin, China

**Keywords:** bacterium-like particles, anchoring protein, surface display platform, multi-epitope antigen, immunogenicity

In the published article, there was an error in the Funding statement. One of the funding sources was inadvertently omitted from the published version. “The author(s) declare financial support was received for the research, authorship, and/or publication of this article. This research was funded by the National Key Research and Development Program of China (2022YFF0710505-2); National Natural Science Foundation of China (31672532); the SIPT Project of Northeast Agricultural University (202310224043S, X202310224172, and X202310224024).” The correct Funding statement appears below.

