# Supplementary material for: A novel bacterium-like particles platform displaying antigens by new anchoring proteins induces efficacious immune responses
Source: Front Microbiol. 2024 May 22;15:1395837. doi: 10.3389/fmicb.2024.1395837 (PMC11150769; doi:10.3389/fmicb.2024.1395837)
Supplement: Supplementary file 1 [file Data_Sheet_1.DOCX]

Supplementary Material

# Supplementary Table 1. PCR primers sequences

| Gene  name | Sequence (5’-3’) | Length(bp) | Reference |
| --- | --- | --- | --- |
| β-actin | F: CCAGTTGGTAACAATGCCATGT | 154 | Shi et al., 2022 |
|  | R: GGCTGTATTCCCCTCCATCG |  |  |
| IL-1β | F: TTCAGGCAGGCAGTATCACTC | 75 | Pan et al., 2022 |
|  | R:GAAGGTCCACGGGAAAGACAC |  |  |
| IL-4 | F: GCCGATGATCTCTCTCAAGTGA | 102 | Shi et al., 2022 |
|  | R:GGTCTCAACCCCCAGCTAGT |  |  |
| IL-6 | F: AAAGAGTTGTGCAATGGCAATTCT | 51 | Shi et al., 2022 |
|  | R:AAGTGCATCATCGTTCATACA |  |  |
| IL-10 | F: CGCAGCTCTAGGAGCATGTG | 105 | Shi et al., 2022 |
|  | R:GCTCTTACTGACTGGCATGAG |  |  |
| IL-12 | F: ACAGCACCAGCTTCTTCATCAG | 75 | Cheng et al., 2014 |
|  | R:TCTTCAAAGGCTTCATCTGCAA |  |  |
| TNF-α | F: CCCCAAAGGGATGAGAAGTT | 132 | Pan et al., 2022 |
|  | R:CACTTGGTGGTTTGCTACGA |  |  |
| IFN-γ | F: AGACAATCAGGCCATCAGCA | 135 | Shi et al., 2022 |
|  | R:TGGACCTGTGGGTTGTTGAC |  |  |
| IL-5 | F: GCAATGAGACGATGAGGCTTC | 75 | Zhang and Yi, 2022 |
|  | R:GCCCCTGAAAGATTTCTCCAATG |  |  |
| TGF-β1 | F: CCACCTGCAAGACCATCGAC | 91 | Chen et al., 2016 |
|  | R:CTGGCGAGCCTTAGTTTGGAC |  |  |
| OACD | F: GAATTCGTCGACGGTGGTGGTTCTCCGGACAACGG | 474 | This study |
|  | R:TCTAGACTCGAGAGCCAGGCAGTC |  |  |
| RFP | F: CATATGGCTAGCGCGTCGTCC | 690 | This study |
|  | R:GAATTCCAGGAACAGGTGATG |  |  |
| Hag | F: GGAATTCCATATGAGAATTAACCACAATATTGCAGCG | 921 | This study |
|  | R:CCGCTCGAGACGTAATAATTGAAGTACGTTTTG |  |  |


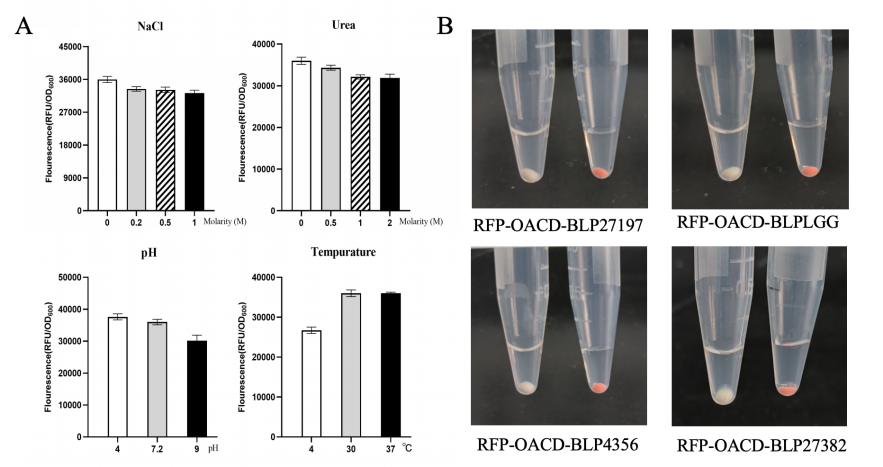


**Supplementary Figure 1** **(A)** Stability detection of the binding product of CPMEA-OACD and BLP23017. The abscissa represents NaCl with different molar concentrations, urea with different molar concentrations, different pH values, and different temperatures. The ordinate is each fusion product was fixed at OD600 of 1 and the RFU of each sample was measured by fluorescence spectrophotometer. Results are the averages from three independent experiments with standard deviations indicated by error bars. **(B)** Binding ability of OACD to different lactic acid bacteria (LAB)*.* The white precipitate of bacteria indicates the RFP did not bind to BLPs. The red precipitate of bacteria indicates the binding products of RFP-OACD with BLPs.


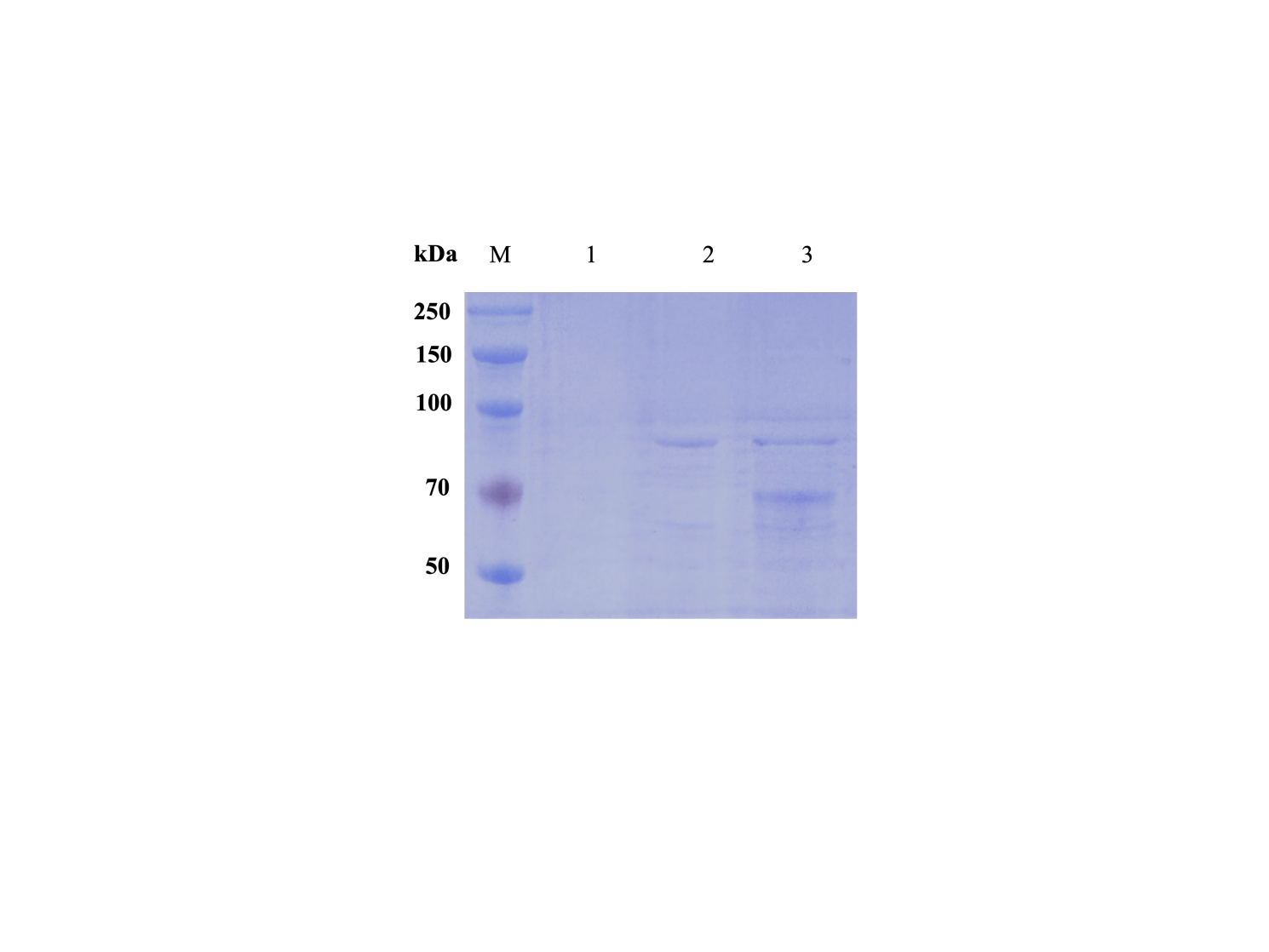


**Supplementary Figure 2.** Identification of the binding product of fusion proteins and BLP23017 by SDS-PAGE. M: Protein Marker, 1: BLP23017, 2: CPMEA-OACD-BLP23017, 3: CPMEA-OACD-Hag-PA-BLP23017.

CPMEA：

ATGACCATCTCTTACGAACAGCCGGACAAAAAAACCATCCAGCGTAAAGACGACAAAAAAAACGGTGACAAAAACTTCACCGACGACCGTGACAAAAAAGGTGAAAAAGACGCTGGTACCAAAAAACTGAAAGACGAAAACCTGAAAATCGACGACAAAAAAATCTCTGGTAACTCTACCAAAAAAGAAATCCGTCGTGACGGTAAAAAAGAATGCAAACAGCGTAACTACAACACCATGAAAAACAAAAAAAACGACAAAAAAGTTACCCTGGCTTGCACCGAATGCAAACAGCGTAACTACAACACCATGAAAAAAGAACAGCGTGAAAACGACGACGTTGTTATGGACAACGGTATCAAAATCAAAAAAATGCTGGACAACTCTGACAAAGACGCTATCCGTATCAAAGCTTGCTCTAAAAAAGACGCTACCCTGGAATACAAAAACACCATCCTGGGTAAAAAATTCAAAATCGCTGCTTACTACTCTATCGGTGGTAACATCTCTGTTGCTGCTTACAACGAATTCATGTTCAAAATCAACTGGGCTGCTTACACCATCTCTTACGAACAGCCGGACTTCGCTGCTTACATCCAGCGTAAAGACGACGCTAACCTGGCTGCTTACAACCTGGCTTCTTGGGACATCAAATTCGCTGCTTACAAACTGAAAGACGAAAACCTGAAAATCGCTGCTTACGCTTACATCTCTACCTCTGGTGAAAAAGCTGCTTACTCTAAAGACACCTACACCTTCAAACTGGCTGCTTACTACCTGAACGACCTGGCTATGAACTACGCTGCTTACATGATGCCGCTGTTCTTCGAATCTAAAGCTGCTTACGAAGTTAACGCTCTGCAGCCGACCCTGGCTGCTTACGAACGTCAGTTCCGTGAACGTGACTTCGCTGCTTACCACGAACTGGGTCACAACTTCGACACCGCTGCTTACCACCTGGCTCAGCTGGCTCCGCTGTGGGCTGCTTACGTTGCTGCTTCTGACGCTATGGAACTGGCTGCTTACGCTATGGAACTGGACCTGACCGAATTCGGTCCGGGTCCGGGTATCGGTGGTAACATCTCTGTTGAAGGTAAAACCGCTGGTACCGGTATCGGTCCGGGTCCGGGTGCTTCTTGGGACATCAAATTCGTTGAAACCAAAGACGGTTACAACGGTCCGGGTCCGGGTTCTGAATACAACGAATTCATGTTCAAAATCAACTGGCAGGACCACGGTCCGGGTCCGGGTGACTACATGTACTTCGGTATCAAAACCAAAGACGGTAAAACCCAGGGTCCGGGTCCGGGTTTCGAATCTAAATACAAAACCAAAACCCGTATCACCGACCAGAACATCTGGGGTCCGGGTCCGGGTCCGAAAGTTGGTCTGGACGACTACTCTAACAACGAACTGTACAACGGTCCGGGTCCGGGTCTGTGGCAGCTGTACCTGTACGACAACACCTTCTACGGTAAATTCGGTCCGGGTCCGGGTAAAGGTGACGGTTTCACCGACAACGCTAAAGTTTCTGTTTCTACCTCTGGTCCGGGTCCGGGTCTGCAGCCGACCCTGTCTGTTAACCCGGTTATCACCCTGGCTCTGGGTGAAGCTGCTGCTAAATCTGAAGACGGTAACAACTTCCGTAAAAAAAAATGGGACGACTCTGGTTCTCTGAAAACCCTGAAATTCAACTCTAAAAAATCTGAAGCTGACCGTGACTACAAAGAAAACGCTGTTGACGGTGACGAAAACACCAAAAAACCGCGTCAGAACTCTCGTAACGGTCACAAAAAACGTCAGGACGCTCTGAACGGTTCTATCTCTAAATACGAAGAA

Hag:

CATATGAGAATTAACCACAATATTGCAGCGCTTAACACACTGAACCGTTTGTCTTCAAACAACAGTGCGAGCCAAAAGAACATGGAGAAACTTTCTTCAGGTCTTCGCATCAACCGTGCGGGAGATGACGCAGCAGGTCTTGCGATCTCTGAAAAAATGAGAGGACAAATCAGAGGTCTTGAAATGGCTTCTAAAAACTCTCAAGACGGAATCTCTCTTATCCAAACAGCTGAGGGTGCATTAACTGAAACTCATGCGATCCTTCAACGTGTTCGTGAGCTAGTTGTTCAAGCTGGAAACACTGGAACTCAGGACAAAGCAACTGATTTGCAATCTATTCAAGATGAAATTTCAGCTTTAACAGATGAAATCGATGGTATTTCAAATCGTACAGAATTCAATGGTAAGAAATTGCTCGATGGCACTTACAAAGTTGACACAGCTACTCCTGCAAATCAAAAGAACTTGGTATTCCAAATCGGAGCAAATGCTACACAGCAAATCTCTGTAAATATTGAGGATATGGGTGCTGACGCTCTTGGAATTAAAGAAGCTGATGGTTCAATTGCAGCTCTTCATTCAGTTAATGATCTTGACGTAACAAAATTCGCAGATAATGCAGCAGATACTGCTGATATCGGTTTCGATGCTCAATTGAAAGTTGTTGATGAAGCGATCAACCAAGTTTCTTCTCAACGTGCTAAGCTTGGTGCGGTACAAAATCGTCTAGAGCACACAATTAACAACTTAAGCGCTTCTGGTGAAAACTTGACAGCTGCTGAGTCTCGTATCCGTGACGTTGACATGGCTAAAGAGATGAGCGAATTCACAAAGAACAACATTCTTTCTCAGGCTTCTCAAGCTATGCTTGCTCAAGCAAACCAACAGCCGCAAAACGTACTTCAATTATTACGTCTCGAG

PA:

ATGGCTGGTAACACCAACTCTGGTGGTTCTACCACCACCATCACCAACAACAACTCTGGTACCAACTCTTCTTCTACCACCTACACCGTTAAATCTGGTGACACCCTGTGGGGTATCTCTCAGCGTTACGGTATCTCTGTTGCTCAGATCCAGTCTGCTAACAACCTGAAATCTACCATCATCTACATCGGTCAGAAACTGGTTCTGACCGGTTCTGCTTCTTCTACCAACTCTGGTGGTTCTAACAACTCTGCTTCTACCACCCCGACCACCTCTGTTACCCCGGCTAAACCGACCTCTCAGACCACCGTTAAAGTTAAATCTGGTGACACCCTGTGGGCTCTGTCTGTTAAATACAAAACCTCTATCGCTCAGCTGAAATCTTGGAACCACCTGTCTTCTGACACCATCTACATCGGTCAGAACCTGATCGTTTCTCAGTCTGCTGCTGCTTCTAACCCGTCTACCGGTTCTGGTTCTACCGCTACCAACAACTCTAACTCTACCTCTTCTAACTCTAACGCTTCTATCCACAAAGTTGTTAAAGGTGACACCCTGTGGGGTCTGTCTCAGAAATCTGGTTCTCCGATCGCTTCTATCAAAGCTTGGAACCACCTGTCTTCTGACACCATCCTGATCGGTCAGTACCTGCGTATCAAA

OACD：

GAATTCGTCGACGGTGGTGGTTCTCCGGACAACGGCATGCTGAGCCTGGGTGTTTCCTACCGTTTCGGTCAGGGCGAAGCAGCTCCAGTAGTTGCTCCGGCTCCAGCTCCGGCACCGGAAGTACAGACCAAGCACTTCACTCTGAAGTCTGACGTTCTGTTCAACTTCAACAAAGCAACCCTGAAACCGGAAGGTCAGGCTGCTCTGGATCAGCTGTACAGCCAGCTGAGCAACCTGGATCCGAAAGACGGTTCCGTAGTTGTTCTGGGTTACACCGACCGCATCGGTTCTGACGCTTACAACCAGGGTCTGTCCGAGCGCCGTGCTCAGTCTGTTGTTGATTACCTGATCTCCAAAGGTATCCCGGCAGACAAGATCTCCGCACGTGGTATGGGCGAATCCAACCCGGTTACTGGCAACACCTGTGACAACGTGAAACAGCGTGCTGCACTGATCGACTGCCTGGCTCTCGAG
