# Supplementary figures and images for: A novel bacterium-like particles platform displaying antigens by new anchoring proteins induces efficacious immune responses
Source: Front Microbiol. 2024 May 22;15:1395837. doi: 10.3389/fmicb.2024.1395837 (PMC11150769; doi:10.3389/fmicb.2024.1395837)

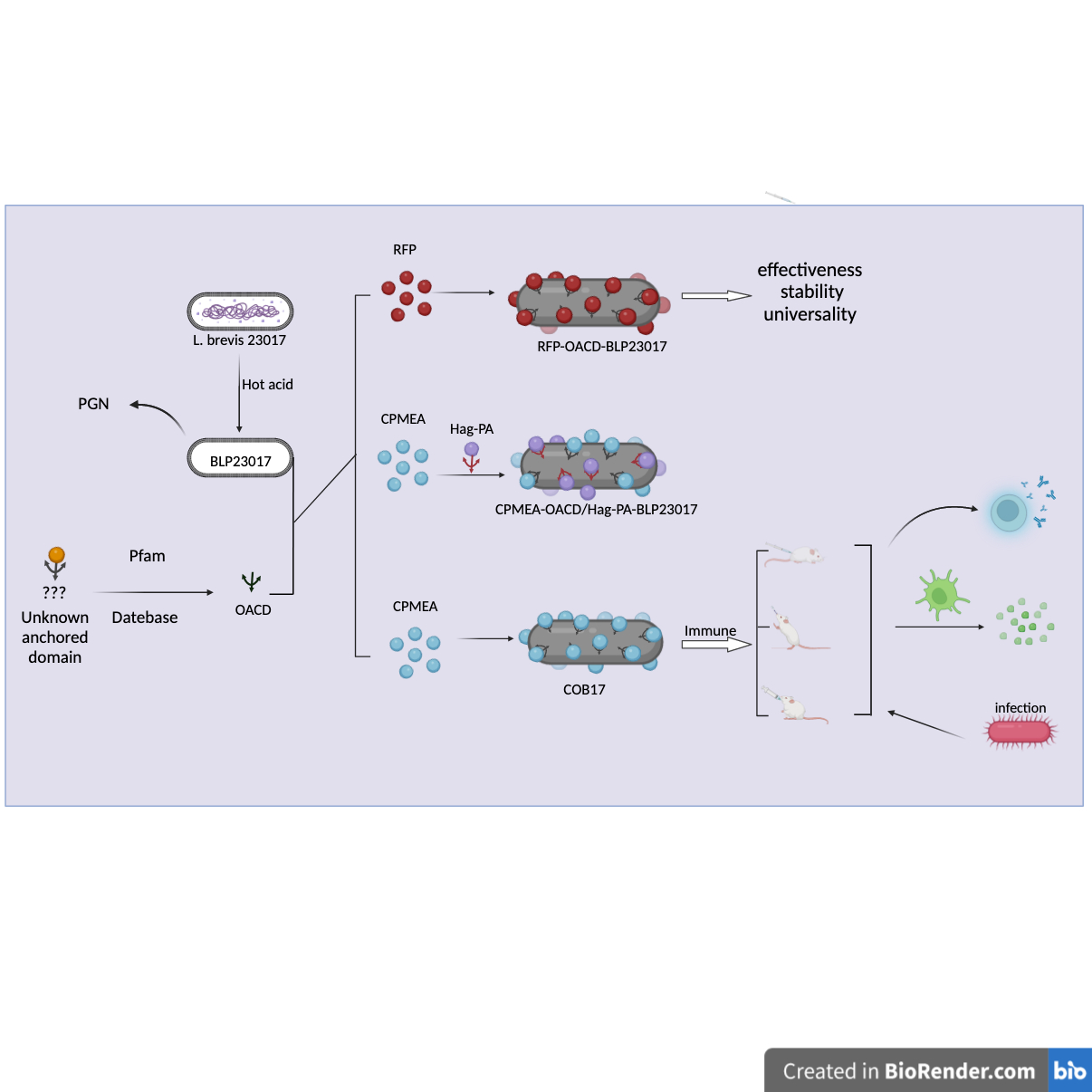

Supplement: Supplementary file 2 [file Image_1.JPEG]
